# A Novel Treatment for Outer Ear Keloids Using Rubber Band Ligation—From Rear to Ear

**DOI:** 10.1002/ccr3.72132

**Published:** 2026-03-17

**Authors:** Johanna Sundén, Krister Tano, Anders Niklasson

**Affiliations:** ^1^ Department of Clinical Science, Division of Otolaryngology, Sunderby Research Unit Umeå University Umeå Sweden; ^2^ Department of Otolaryngology Karolinska University Hospital Stockholm Sweden

**Keywords:** human, keloid, outer ear, treatment

## Abstract

This study demonstrates the use of a rubber band ligation for treating outer ear keloids. The method is effective, safe, and well‐tolerated by the patients. Furthermore, it is inexpensive, does not require surgical facilities, surgical skills, or extensive medical training, and relies only on basic equipment. Therefore, it is accessible to healthcare systems with varying resource levels.

## Graphical abstract

We present a novel treatment for outer ear keloids using rubber band ligation in 3 patients.

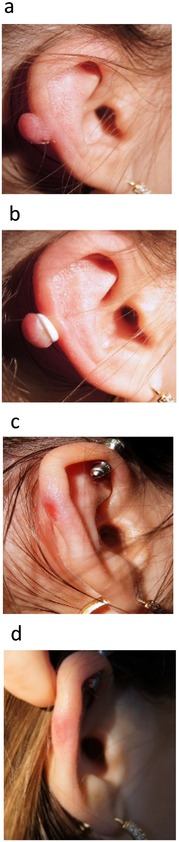

## 1 Introduction

A keloid is an abnormal scarifications of the skin that only occurs in humans [1] and often develops after trauma or surgery. The main clinical trait that differentiates keloids from hypertrophic scars is that the keloids grow beyond the original trauma in the skin. Keloids usually do not subside over time, often tend to worsen after surgery, and may not start to grow until months after trauma or surgery. In contrast, hypertrophic scars usually decrease over time, are less recurrent, and usually appear soon after surgery [2]. Furthermore, histological differences are evident; keloids consist of an irregular collagen pattern, as opposed to hypertrophic scars where the collagen is parallel to the epithelial surface [1, 2].

The outer ear is the most common site of keloid formation [3]. The chest, shoulder, and neck are other predisposed locations of keloid formation [1]. Approximately 2.5% of the ear piercings are complicated by keloid formation. Risk factors for developing keloids of the outer ear include puberty, pregnancy, genetic predisposition, and pigmented skin [1], in contrast to individuals with albinism, who do not seem to develop keloids [4]. Keloids usually develop at the ages of 10–30 years, and there is no difference between gender [4]. Except for cosmetic concerns, keloids may cause symptoms such as itching, pain, tension, and hyperesthesia [2].

Different treatment strategies have been tried throughout history with varying results. Methods used as single or multi‐modal treatments are: excision in combination with local injection of steroids [5], radiation therapy [6], freezing [7], injection of 5‐fluorouracil [8] or interferon [9], pressure [10, 11], silicone dressings [2], laser therapy [12, 13], and topical mitomycin C [14]. A similar treatment as in the present study is repeated strangulation of the keloid with a ligature [15]. Although multimodal treatments show less recurrence rate [16, 17] all treatments show recurrences in various degrees, especially over a long time [1].

## 2 Case History/Examination

The aim of the present study was to treat keloids of the outer ear with the use of a rubber band, instead of repeated strangulation with a ligature. Furthermore, we wanted to evaluate this method regarding patient satisfaction, side effects and recurrences. Inclusion criteria were age > 18 years and a keloid with a pedicle less than 15 mm in diameter in order to be able to fit the rubber band around the pedicle.

## 3 Differential Diagnosis, Investigations and Treatment

**FIGURE 1:**
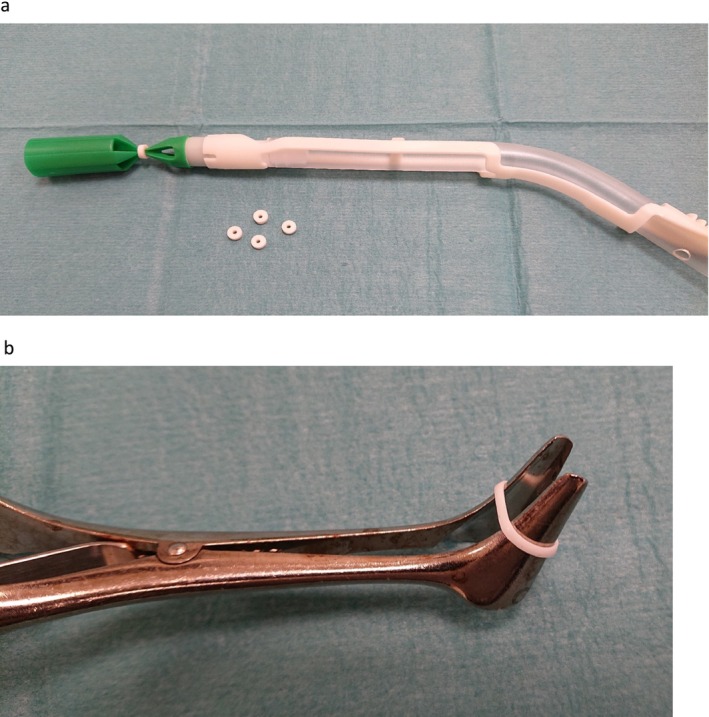
(a) The application device (KilRoid). (b) Nasal speculum with the rubber band.

Our treatment method consisted of the application of a rubber band, commonly used for the treatment of hemorrhoids (KilRoid, Mediplast AB, Sweden) around the pedicle of the keloid which typically detached spontaneously after approximately 2 weeks due to ischemic necrosis (Figure 1). The mechanism resembles repeated ligature strangulation, but the elasticity of the rubber band provides continuous compression without the need for retightening.

**FIGURE 2:**
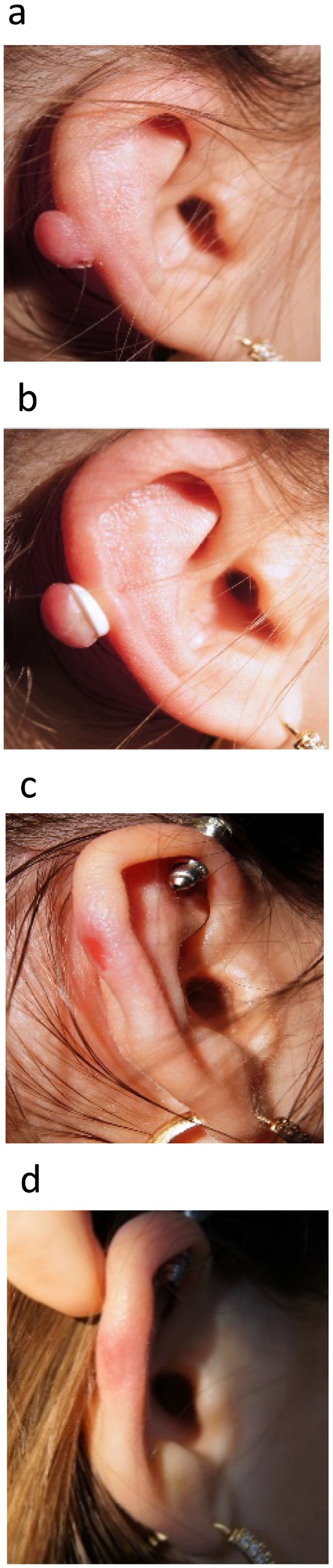
Photos of patient no 2 at different time intervals: (a) Before treatment. (b) After application of the rubber band. (c) After 2 weeks. (d) After 1 year with no sign of recurrence.

All included patients gave their informed consent before treatment. Three patients were treated with the rubber band (Figure 2). However, one of the patients (no 3) had a keloid which was initially too big for the rubber band, but after local injection of corticosteroids (triamcinolon acetonide 40 mg/mL) and pressure treatment for 1 month, the keloid was soft enough to fit a rubber band over the pedicle.

**FIGURE 3:**
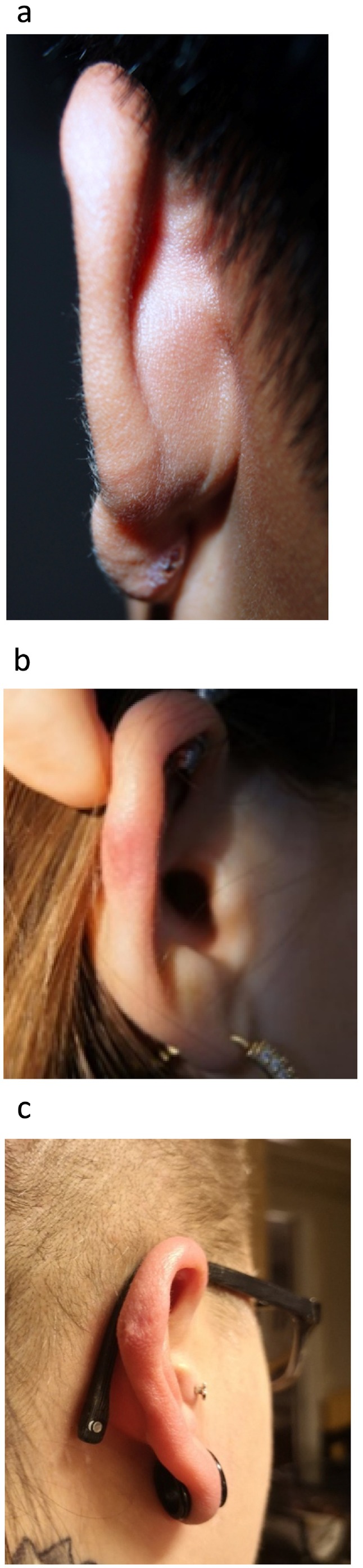
Photos of the final results of the included patients. (a) Pat no 1 (2 years), (b) Pat no 2 (1 year), (c) Pat no 3 (1.5 years).

At the first visit, the included patients received the rubber band either with the applicator or with a nasal speculum if the keloid had a diameter of more than 10 mm. Patients were scheduled for follow‐up visits with photographic documentation at 2 and 4 weeks, and at 6 and 12 months (Figure 3). Two patients were female and one male, had non‐pigmented skin, and had a keloid at the helix of the outer ear.

## 4 Conclusion and Results

Overall, the patients were satisfied with the treatment. The symptoms reported were mostly initial tenderness. One of the patients reported some pain during the first 2 days but still completed the treatment. The cosmetic end results were good or satisfactory at a mean follow‐up of 18 months (range 12–36 months); no major complications were observed. The results were documented and presented as pictures (Figure 3). The present study shows that it is possible to remove keloids of the outer ear at any out‐patient clinic, without any operating facilities. The rubber band method is fast, well tolerated, and inexpensive, producing satisfactory results without major complications.

## 5 Discussion

This study aimed to evaluate a novel method for treating outer ear keloids using a rubber band ligation. The technique was adapted from an established method of removing hemorrhoids with repeated ligature strangulation. Above we present the first three cases treated with this method. We have continued to treat patients with the same procedure in 10 patients total, with equally good results. Some patients experienced mild discomfort during the first 1–2 days, and overall cosmetic outcomes were good. The rubber band ligation therapy was used successfully as a single treatment. Recurrence after 3 months was seen in one individual but was successfully treated with local steroid injections. Postoperative intralesional injections with corticosteroids have demonstrated good results in previous studies. Therefore, a follow‐up of the patients is important, and if signs of recurrence are noticed, intralesional injection with corticosteroids could be an option.

The postoperative intralesional corticosteroid injection has also been used successfully in combination with pressure to treat keloids. Furthermore, silicon dressing has been shown to be beneficial in the treatment of keloids; thus, the pressure preferably could be complemented with silicon sheeting.

The main limitation of the present study is the lack of comparison with other methods, but the aim was primarily to investigate if this rubber band treatment of outer ear keloids is safe, effective, and tolerable for the patients. Furthermore, we did not use local corticosteroids in a systematic way, but this is something that could be studied in the future. A future multimodal approach with post‐ligation treatment including radiation therapy or pressure could also be explored in the future. Both of which have shown good postoperative advantages after surgery. Although limited by its small sample size and lack of control group, this pilot study suggests that rubber band ligation may be a feasible and resource‐efficient option for selected auricular keloids.

The rubber band technique showed no major side effects and was easy to perform. Some patients suffered minor pain when the ligation band was applied but all but one experienced that it was not a major issue. This discomfort could be solved in the future by using rubber bands with different elasticity according to the size of the keloid. Future ligation bands may also be made of silicone, which might promote even less recurrence.

## Author Contributions

**Johanna Sundén:** investigation, writing – original draft. **Krister Tano:** methodology, supervision. **Anders Niklasson:** methodology, supervision, validation.

## Funding

The authors have nothing to report.

## Ethics Statement

Ethical permission for the study was granted by the regional ethical committee in Umeå (DNR: 2016/19‐31). Permission was also granted from the head of the department to use the medical device (rubber bands originally designed for hemorrhoid ligation) for an off‐label external use on the outer ears.

## Consent

Written informed consent from the patient was obtained according to journal guidelines.

## Data Availability

The data that support the findings of this study are available upon request from the authors—anonymized.
